# Exploring behavioural motivations of treatment refusal in cancer: a Q-methodological approach

**DOI:** 10.1007/s00520-025-09666-5

**Published:** 2025-07-08

**Authors:** Ruby-Koyllor A. Gleeson, Nicolas H. Hart, Darren Haywood, Frank D. Baughman

**Affiliations:** 1 School of Population Health, Faculty of Health Sciences, Curtin University, Bentley, WA 6102 Australia; 2 Human Performance Research Centre, INSIGHT Research Institute, Faculty of Health, University of Technology Sydney (UTS), Sydney, NSW Australia; 3 Caring Futures Institute, College of Nursing and Health Sciences, Flinders University, Adelaide, SA Australia; 4 Exercise Medicine Research Institute, School of Medical and Health Sciences, Edith Cowan University, Perth, WA Australia; 5 Cancer and Palliative Care Outcomes Centre, Faculty of Health, Queensland University of Technology (QUT), Brisbane, QLD Australia; 6 Institute for Health Research, University of Notre Dame Australia, Perth, WA Australia; 7 Department of Mental Health, St. Vincent’s Hospital Melbourne, Fitzroy, VIC Australia; 8 Department of Psychiatry, Melbourne Medical School, Dentistry and Health Sciences, University of Melbourne, Parkville, VIC Australia; 9 Centre for Mental Health and Brain Sciences, Swinburne University of Technology, Hawthorn, VIC Australia

**Keywords:** Treatment refusal, Alternative medicine, Cancer, Oncology

## Abstract

**Background:**

Cancer treatment refusal is known to lower survival rates and increase cancer symptoms in individuals with cancer. Behavioural motivations of treatment refusal need to be elucidated for better cancer care. Using Q-methodology, a mixed methods research approach, we explored behavioural motivations of treatment refusal in individuals diagnosed with cancer.

**Method:**

Thirty-nine individuals (*n* = 39; age = 49.2 ± 12.2 years) were recruited from Australia, the UK, and the USA, who had refused cancer treatment within the past decade. Participants completed an online demographic questionnaire and a Q-sort activity which required the organisation and ranking of 44 statements on potential treatment refusal motivations. Q-sort data were analysed with an inverted factor analysis. Compositive Q-sorts, distinguishing statements, and demographic data facilitated interpretation of the resulting factors.

**Results:**

Eight factors, accounting for 66% of total variance, were identified and interpreted. The eight factors were as follows: (1) I was not motivated by my health status, (2) Treatment was too risky for how unwell I was, (3) I was motivated by my age, (4) I did not distrust the medical system and practitioners, (5) Religious and spiritual practices would heal me, (6) I was influenced by my religious and spiritual beliefs, (7) My prognosis was not good enough, and (8) I wanted to heal naturally.

**Conclusion:**

Diverse motivations for cancer treatment refusal were found, which could help practitioners understand an individual’s considerations regarding treatment decisions. Future research should investigate motivations underpinning cancer treatment refusal and establish person-centred strategies to address concerns when promoting evidence-based cancer treatment.

**Supplementary Information:**

The online version contains supplementary material available at 10.1007/s00520-025-09666-5.

## Introduction

One in five people will develop cancer [1], most of whom receive conventional cancer treatments recommended by medical practitioners [2], such as surgery, hormone therapy, chemotherapy, radiotherapy, immunotherapy, stem cell transplant, and targeted therapy [3]. At its most critical, treatment decisions confront individuals with questions about their own mortality [4]. Given that treatment is typically framed as the primary option to prolong the life of people with cancer, it can be difficult for others to understand why some individuals refuse clinically recommended evidence-based treatment [4]. Findings from studies investigating treatment refusal can be difficult to interpret, partly due to the adoption of varying definitions of treatment refusal. For some, treatment refusal constitutes the rejection of all conventional treatments recommended by medical practitioners, while for others it may involve the rejection of a selection of conventional treatments recommended by medical practitioners [5].

Refusal of cancer treatment has repeatedly been shown to lower survival rates and increase the likelihood of adverse cancer-related symptoms, metastasis, and cancer recurrence [6, 7]. Individuals with stage I pancreatic cancer who underwent surgery had a significantly higher 1-year survival rate than those who refused surgery (74.1% vs. 28.3%, *p* < 0.001; [8]). Surprisingly, there appears to be no consensus on basic estimates, such as how many individuals diagnosed with cancer opt to refuse treatment and follow alternative pathways. The highest treatment refusal rate identified in the literature was 20% for chemotherapy refusal among older women (≥ 65 years old) in the USA diagnosed with high-genomic-risk, oestrogen receptor-positive breast cancer [9]. The lowest treatment refusal rate we identified was 0.2% for surgery refusal among individuals diagnosed with endometrial cancer in the USA [2].

The literature shows various demographic characteristics such as older age, female sex, black ethnicity, unmarried status, and lack of health insurance are associated with an increased treatment refusal risk [10, 11]. Distrust in medical practitioners, uncertainty about treatment benefits, and the desire to avoid long hospital stays provide other potential motivators for cancer treatment refusal; however, these remain untested [12]. While identifying characteristics of individuals more at risk of treatment refusal is helpful for practitioners, these characteristics do not explain the motivations behind treatment refusal [13]. A previous study by Khankeh et al. [14] used a qualitative grounded theory approach to evaluate why patients with cancer refused treatment from the perspectives of patients themselves (*n* = 12), their caregivers (*n* = 5), and health professionals (*n* = 5), finding four key categories; Encounter with Cancer, Fighting Cancer, Coping with Cancer, and Losing Resilience in the Course of Cancer. However, there is a significant gap in the literature regarding the specific behavioural motivations of treatment refusal in people with cancer. Practitioners could offer more tailored support if they understood the behavioural motivations of individuals who consider treatment refusal, which could improve cancer care and cancer outcomes [13].

Cancer treatment refusal studies typically adopt a quantitative approach [12], however, to enable better insights with more sensitive and comprehensive analyses, a mixed methods approach such as Q-methodology could be used to reveal diverse perspectives about treatment refusal in a systematic and reliable way [15]. As such, the aim of this study was to utilise Q-methodology to explore the behavioural motivations of treatment refusal in individuals diagnosed with cancer.

## Method

### Study design

A mixed methods approach using Q-methodology was adopted [15]. Statements for use within the Q-Sort task were developed by the researchers via a review of the treatment refusal literature [16]. Participants then engaged in qualitative sorting during the Q-Sort task, assessed by the researchers through quantitative analysis and qualitative interpretation [17].

### Participants

Purposive sampling was used to recruit participants [18], who were required to be 18 years or older at the time of testing, and have refused at least one conventional cancer treatment recommended by a medical practitioner in the past decade. The treatment refusal time range was chosen to capture and consider the dramatic changes to conventional cancer treatment over time [19]. Participants in Australia, the UK, or the USA were recruited. Participants were recruited via the participant recruitment platform Prolific (https://www.prolific.com). Prolific is a valid and reliable online platform where researchers recruit eligible participants in exchange for remuneration and has been used for wide ranging behavioural research including within oncology [20–25].

### Measures

#### Demographic questionnaire

An online survey platform, Qualtrics (https://www.qualtrics.com), was used to deliver the demographic questionnaire, which consisted of 14 items collecting sociodemographic information and information specific to participants’ last experience refusing cancer treatment.

#### Q-Sort

**Fig. 1:**
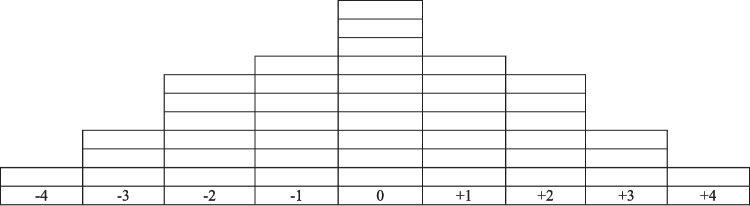
Q-Sort task grid.Note: This figure shows the sorting grid presented to each participant. Each grid box represents a space where a Q-sample statement can be placed. − 4 = Most uncharacteristic statement; − 3 = Very uncharacteristic statements; − 2 = Moderately uncharacteristic statements; − 1 = Mildly uncharacteristic statements; 0 = Neutral statements; + 1 = Mildly characteristic statements; + 2 = Moderately characteristic statements; + 3 = Very characteristic statements; 4 = Most characteristic statement

The Q-Sort task consisted of the Q-sample (comprised 44 statements), each representing a potential behavioural motivation for refusing cancer treatment; and the sorting grid (Fig. 1), resembling a quasi-normal distribution [18]. Participants completed the Q-Sort online by arranging each Q-Sample statement on the sorting grid according to how characteristic the statement was of their last treatment refusal.

### Procedure

#### Q-Sort generation

Following ethics approval from Curtin University’s Human Research Ethics Committee (HRE2024-0359), the concourse was developed to include statements that contained potential cancer treatment refusal motivations, through reviewing social media discourse and relevant studies. Social media discourse was first-hand accounts of cancer treatment refusal from Facebook, Reddit, and the webpage forums of various cancer foundations. Studies were sourced from ProQuest, Scopus, and Web of Science, using the search term “cancer treatment refusal”. When further data collection no longer contributed new insights into the research topic, saturation was achieved and the concourse was completed [26]. The final concourse contained 118 statements.

The Q-sample (*n* = 44) was developed through extensive deliberation and discussion about choosing statements that best reflected the concourse’s diversity. The recommendation that a Q-sample has between 30 and 60 statements was also considered [27]. The Q-sample and sorting grid were combined and transformed into an online Q-sort through JavaScript. The demographic questionnaire, the Ten-Item Personality Inventory (TIPI; [28]), and the Internal–External Locus of Control (IE-4; [29]) were inputted into an online survey hosted on Qualtrics. Participant responses to the TIPI and IE-4 were collected for a separate study. A link to the Q-sort was embedded into the online survey.

#### Data collection

Participants were recruited through the Prolific platform and received $7.25 AUD for remuneration upon completion. Participants accessed the online survey via a link provided in the study’s advertisement. All participants were required to complete a ReCAPTCHA that screened for bots, and were then directed to complete the demographic questionnaire, TIPI, and IE-4 questionnaires. Afterward, participants were redirected to a separate website where they were presented with the Q-sort and its instructions. After participants completed the Q-sort, their participation in the study ended.

### Analysis

Completed Q-sorts were analysed using an inverted factor analysis (IFA; [18]), thereby grouping Q-sorts by participants’ shared viewpoints [27]. To run the IFA, Q-sort data were uploaded onto KenQ Analysis Desktop Edition (KADE) v1.3.1 [30], where Pearson correlation coefficients between the Q-sorts were produced. A principal components analysis (PCA) was then run on KADE to extract factors [31]. All factors extracted were retained and rotated using varimax rotation [32]. No manual sorting occurred at any stage. Descriptive statistics analysis was conducted on the demographic questionnaire data.

## Results

Forty-three participants were recruited, consistent with prior Q-methodology studies [33, 34]. Four participants from Prolific were ineligible for inclusion due to their treatment refusal being more than 10 years ago (*n* = 3) or concerns about their data quality (*n* = 1). The final P-set consisted of 39 participants (*M*_age_ = 49.2 years, *SD* = 12.2). Twenty-five participants identified as female (64.1%) and 14 as male (35.9%). Ethnicities among the P-set were White American (25.6%), White European (23.1%), Black or African American (12.8%), Hispanic (12.8%), Non-Indigenous Australian (7.7%), mixed ethnicity (5.1%), Aboriginal and/or Torres Strait Islander (2.6%), and African (2.6%). Three participants did not disclose their ethnicity (7.7%). At the time they refused treatment, the P-set’s mean age was 45.4 years (*SD* = 12.3). Further sociodemographic characteristics of the P-set, at the time participants refused treatment, are reported in Table 1. Specific cancer characteristics of the P-set are shown in Table 2.

**Table 1:** Sociodemographic characteristics of the P-set at the time of cancer treatment refusal

Characteristic	P-set	
	*n*	*%*
Age		
< 18	1	2.6
25–34	7	17.9
35–44	11	28.2
45–54	8	20.5
55–64	11	28.2
≥ 65	1	2.6
Location		
USA	25	64.1
UK	9	23.1
Australia	5	12.8
Employment status		
Employed	29	74.4
Unemployed	2	5.1
Student	1	2.6
Retired	5	12.8
Unable to work	2	5.1
Highest educational level		
Secondary education	6	15.4
Some college, no degree	8	20.5
Post-secondary, non-tertiary education	3	7.7
Bachelor’s degree	9	23.1
Master’s degree	12	30.8
Doctoral degree	1	2.6
Household income in Australian dollars		
Less than $20,000	2	5.1
$20,000–$39,999	9	23.1
$40,000–$59,999	3	7.7
$60,000–$79,000	3	7.7
$80,000–$99,000	5	12.8
$100,000–$119,999	6	15.4
$120,000–$139,999	4	10.3
$140,000–$159,999	3	7.7
$160,000–$179,999	1	2.6
≥ $180,000	2	5.1
Prefer not to say	1	2.6
Marital status		
Divorced	3	7.7
Single	14	35.9
Married	22	56.4

**Table 2:** Characteristics of the P-set specific to cancer

Characteristic	P-set	
	*n*	*%*
Cancer type^a^		
Adrenal	1	2.6
Breast	9	23.1
Cervical	2	5.1
Colorectal	2	5.1
Endometrial	2	5.1
Kidney	2	5.1
Leukaemia	1	2.6
Lung cancer	2	5.1
Non-Hodgkin’s lymphoma	1	2.6
Prostate	5	12.8
Skin	7	17.9
Soft tissue sarcoma	1	2.6
Thyroid	2	5.1
Uterine	2	5.1
Cancer stage^a^		
Stage I	20	51.3
Stage II	7	17.9
Stage III	7	17.9
Stage IV	4	10.3
Not sure/prefer not to say	1	2.6
Conventional cancer treatment refused^a^		
Chemotherapy	16	61.5
Hormone therapy	5	33.3
Immunotherapy	3	27.3
Radiotherapy	13	54.2
Stem cell treatment	1	25.0
Surgery	10	30.3
Targeted therapy	3	33.3
Conventional cancer treatment received^b^		
Chemotherapy	10	25.6
Hormone therapy	10	25.6
Immunotherapy	8	20.5
Radiotherapy	11	28.2
Stem cell transplant	3	7.7
Surgery	23	59.0
Targeted therapy	6	15.4
None	5	12.8
Received alternative or complementary treatments^b^		
Yes	11	28.2
No	28	71.8
Cancer status^c^		
In remission	13	33.3
Cancer-free after treatment	11	28.2
Undergoing active treatment	5	12.8
Undergoing further diagnosis	4	10.3
Cancer recurrence	3	7.7
Did not disclose	2	5.1
Palliative care	1	2.6

^a^Answers represent the time participants last refused conventional cancer treatment. ^b^Answers represent whether participants received the treatment at some point in time. ^c^Answers represent participants’ status at the time of testing

### Factor analysis

Eight factors were identified by the PCA (eigenvalue ≥ 1), which accounted for 67% of the total variance. The total variance was > 60%, so our PCA model was considered optimal [35]. Following the eigenvalue criterion that considers a factor with an eigenvalue ≥ 1 significant enough for further interpretation, all eight factors were retained and rotated using varimax rotation [32]. After rotation, the eight factors accounted for 66% of the total variance. Statistics about each factor are shown in Table 3. Each factor had a composite reliability > 0.80. Thus, all factors were considered reliable [35].

**Table 3:** Factor characteristics

Factor	*N* of defining Q-sorts	Composite reliability	% explained variance
1	6	0.96	9
2	4	0.94	8
3	3	0.92	7
4	5	0.95	8
5	4	0.94	8
6	4	0.94	6
7	8	0.97	12
8	5	0.95	8

Composite Q-sorts, representing the Q-sort layout most representative of each factor, were produced [34]. As shown in Table 4, the distinguishing statements of each factor were the statements with significantly different (*p* < 0.005) placements on the sorting grid, which were specific to certain factors [34]. No consensus statements—statements with an agreed-upon ranking across all Q-sorts—were identified [35]. Table 5 describes the motivations for treatment refusal of the participants whose Q-sort defined each factor.

**Table 4:** Distinguishing statements of each factor

Factor	Statement	*z*-score
1	10. I believed that my body could heal itself without the need for conventional cancer treatment	0.06*
	5. I believed that conventional cancer treatment would be detrimental to my mental health	− 1.04**
	4. I believed that conventional cancer treatment would be detrimental to my emotional health	− 1.8**
	3. I believed that conventional cancer treatment was not tailored to my individual needs	− 1.92**
	2. I believed my other medical conditions made conventional cancer treatment too risky	− 2.29**
	1. I believed my poor health made conventional cancer treatment too risky	− 2.92*
2	1. I believed my poor health made conventional cancer treatment too risky	0.97**
	30. I was concerned about the potential long-term side effects of conventional cancer treatment	− 0.82*
	36. I was influenced by stories of people who had bad experiences with conventional cancer treatment	− 1.35*
	41. My cultural beliefs influenced my decision to decline conventional cancer treatment	− 1.9*
3	9. I believed that my age made conventional cancer treatment less beneficial and/or unnecessary	1.85**
	16. I did not trust the medical system, including the practitioner that recommend I underwent conventional cancer treatment	1.1*
4	20. I had seen others undergo conventional cancer treatment before, and I did not want to experience what they had	− 0.96**
	16. I did not trust the medical system, including the practitioners that recommended I underwent conventional cancer treatment	− 1.88*
5	12. I believed that participating in spiritual practices would heal my cancer	1.57**
	11. I believed that participating in religious practices would heal my cancer	1.46**
	26. I wanted to have more control over my treatment decisions	− 0.28**
6	43. My religious beliefs influenced my decision to decline conventional cancer treatment	1.43**
	44. My spiritual beliefs influenced my decision to decline conventional cancer treatment	1.43*
	33. I was concerned that my body would change forever if I underwent conventional cancer treatment	1.2*
	21. I valued quality of life over quantity of life	0*
7	13. I believed that the prognosis I was given if I was to have conventional cancer treatment was not good enough for me to choose to do it	1.96**
	9. I believed that my age made conventional cancer treatment less beneficial and/or unnecessary	− 0.66**
	11. I believed that participating in religious practices would heal my cancer	− 1.73*
	10. I believed that my body could heal itself without the need for conventional cancer treatment	− 2.2**
8	24. I wanted to heal the cancer in my body naturally	1.6**
	12. I believed that participating in religious practices would heal my cancer	− 2.28*

*p < 0.05, **p < 0.01

**Table 5:** Factor Descriptions and Examples

Factors	Factor description	Statements
Factor 1. I was not motivated by my health status (*n* = 6)	Participants were significantly unmotivated to refuse treatment because of their health status They were most motivated by the belief that treatment side effects would reduce their quality of life	“I believed my poor health made conventional cancer treatment too risky”. (Ranked “most uncharacteristic”) “I believed the side effects of conventional cancer treatment would significantly reduce my quality of life”. (Ranked “most characteristic”)
Factor 2. Treatment was too risky for how unwell I was (*n* = 4)	Participants believed their ill health made treatment too risky for them to decide to undergo treatment They were strongly motivated to refuse treatment by their distrust of the medical system and practitioners also	“I believed my poor health made conventional cancer treatment too risky”. (Ranked “moderately characteristic”) “I did not trust the medical system, including the practitioners that recommended I underwent conventional cancer treatment”. (Ranked “most characteristic”)
Factor 3. I Was Motivated by My Age (*n* = 3)	Participants refused treatment because they believed their age made treatment less beneficial or necessary	“I believed that my age made conventional cancer treatment less beneficial and/or unnecessary”. (Ranked “very characteristic”)
	They were strongly motivated to refuse treatment by the desire to avoid impacting their loved ones also	“I did not want my loved ones to have to support me throughout conventional cancer treatment”. (Ranked “most characteristic”)
Factor 4. I did not distrust the medical system and practitioners (*n* = 5)	Participants’ treatment refusal was significantly less motivated by distrust in the medical system and practitioners	“I did not trust the medical system, including the practitioners that recommended I underwent conventional cancer treatment”. (Ranked “most uncharacteristic”)
	Instead, these participants expressed more concern over the financial impact of treatment	“I was concerned about the financial burden of conventional treatment”. (Ranked “moderately characteristic”)
Factor 5. Religious and spiritual practices would heal me (*n* = 4)	Participants were more motivated to refuse treatment because they believed religious and spiritual (R/S) practices would heal their cancer	“I believed that participating in religious practices would heal my cancer”. (Ranked “moderately characteristic”) “I believed that participating in spiritual practices would heal my cancer”. (Ranked moderately characteristic)
Factor 6. I was influenced by my religious and spiritual beliefs (*n* = 4)	Participants reported that they were influenced to refuse treatment by their R/S beliefs However, they had not been influenced by the belief that R/S practices would heal their cancer	“My religious beliefs influenced my decision to decline conventional cancer treatment”. (Ranked “very characteristic”) “I believed that participating in religious practices would heal my cancer”. (Ranked “moderately uncharacteristic”)
Factor 7. My prognosis was not good enough (*n* = 8)	Participants were most motivated by their belief that the prognosis they were given was not good enough to justify them receiving treatment These participants strongly valued quality over quantity of life	“I believed that the prognosis I was given if I was to have conventional cancer treatment was not good enough for me to choose to do it”. (Ranked “most characteristic”) “I valued quality of life over quantity of life”. (Ranked “very characteristic”)
Factor 8. I wanted to heal naturally (*n* = 5)	Participants were significantly motivated to refuse treatment by a desire to heal their cancer naturally They reported the desire to undergo alternative treatments having impacted their refusal also	“I believed that I could manage my cancer through diet and lifestyle"(Ranked “most characteristic”) “I wanted to undergo alternative treatments rather than conventional ones” (Ranked “moderately characteristic”)

*n* represents the number of participants who defined each factor.

## Discussion

Several behavioural motivations for cancer treatment refusal were evident. Eight distinct factors were identified, representing unique motivations for treatment refusal. Factor 1, “I was not motivated by my health status”, represents participants whose refusal of treatment was not driven by how ill they were, but by a desire to preserve their QoL. In the context of cancer, QoL encompasses an individual’s general health and the presence of cancer-related symptoms [36]. Factor 1 participants expressed concern that side effects of chemotherapy, radiotherapy, and targeted therapy would compromise their QoL. Common side effects of cancer treatment include fatigue, vomiting, and nausea [37]. Our findings align with previous studies that reported how individuals with cancer often worry that treatment side effects will negatively impact their QoL [25, 38–40], which are well-founded, as cancer treatment side effects do significantly diminish an individual’s QoL [37, 40–44]. Factor 1 findings extend previous research by highlighting how some individuals with cancer perceive the relationship between QoL and treatment side effects, and how this perception can motivate them to refuse cancer treatment. Furthermore, the experience of Factor 1 participants demonstrates how treatment refusal can be motivated by the anticipated impact of cancer treatment on their lives, rather than the current impact of their cancer.

Factor 2, “Treatment was too risky for how unwell I was”, represents participants who perceived conventional treatment as “too risky” for their health. It is understood that practitioners will not recommend certain cancer treatments if they believe an individual is too unwell to receive them [45]. The experience of individuals who believe themselves too unwell for practitioner-recommended treatment is less understood. Our findings suggest that these individuals are also motivated to refuse treatment because of their distrust of the medical system and the practitioners recommending treatment. This contrasts with the results of Dean et al. [46], who found an association between distrust of the medical system and cancer treatment refusal but not between practitioner distrust and refusal. Our results diverge from this past research, suggesting that some individuals who refuse treatment may distrust both the medical system and its practitioners. Based on our findings, we propose that the distrust some individuals with cancer have towards practitioners may decrease their trust in practitioner recommendations, potentially increasing their likelihood of treatment refusal.

Factor 3, “I was motivated by my age”, describes participants who were motivated to refuse treatment because they believed their age made treatment less necessary or beneficial. At the time of treatment refusal, these participants had an average age (*M*_age_ = 61.2 years, *SD* = 5.8) older than the average age of participants in this study (*M*_age_ = 45.4 years, *SD* = 12.32). Previous studies report that increasing age correlates with treatment refusal [12]. The results of Factor 3 build upon previous studies, suggesting that age does not merely correlate with treatment refusal but can actively motivate it.

Factor 4, “I did not distrust the medical system and practitioners”, represents participants whose treatment refusal was not motivated by distrust in the medical system or practitioners. Previous research has shown that trust in practitioners increases satisfaction with cancer care among individuals with cancer [47]. As this study did not assess trust levels, we cannot comment on the exact level of trust these participants had in the medical system and practitioners. Treatment refusal in these participants was partly motivated by concern of the financial burden of cancer treatment. This concern may reflect “financial toxicity”, which is the financial suffering cancer treatment causes individuals with cancer and their families [48–53]. Most Factor 4 participants refused treatment in the USA (80%), where financial toxicity is common due to the absence of a national insurance scheme [54]. In the USA, a protective factor against financial toxicity is private insurance [54]. The absence of a national insurance scheme and participants’ insurance statuses may have influenced the financial concerns participants expressed; however, this cannot be definitively determined from our data.

Factors 5 and 6, “Religious and spiritual practices would heal me”, and “I was influenced by my religious and spiritual beliefs”, represent participants whose treatment refusal was motivated by R/S. Participants in these factors were either motivated by R/S beliefs or R/S practices, demonstrating the varied roles R/S plays in the lives of individuals with cancer [55]. Research has confirmed that R/S is associated with better patient-reported physical health among individuals with cancer, though R/S is not considered a cancer cure [56]. Our findings that R/S can motivate treatment refusal support previous studies suggesting R/S influences treatment decisions among some individuals with cancer [55]. We did not identify the specific aspects of R/S beliefs or practices that motivate treatment refusal. A prior study found that some individuals refuse cancer treatment based on their belief that R/S would lead them to the best outcome [57].

Factor 7, “My prognosis was not good enough”, represents participants who perceived their prognosis as “not good enough” to justify receiving cancer treatment. Practitioners communicate prognostic information to individuals with cancer to assist them in making informed treatment decisions [58]. Factor 7 participants were also motivated to refuse treatment due to valuing quality over quantity of life. This motivation aligns with the finding Cartwright et al. [59], who reported that some individuals with cancer refuse treatment after considering their prognosis and their value for quality over quantity of life. All participants in Factor 7 identified as female. We were unable to find studies addressing gender differences in cancer prognosis interpretation. In future research, researchers may wish to explore whether more females with cancer are motivated to refuse treatment based on their prognosis than individuals of other genders.

Factor 8, “I wanted to heal naturally”, represents participants whose treatment refusal was motivated by their desire to heal their cancer naturally. These participants believed they could manage their cancer through diet and lifestyle. While diet and lifestyle can reduce cancer risk, no evidence suggests diet and lifestyle can cure cancer [60]. Despite wanting to pursue alternative methods of treatment at treatment refusal, only 40% of Factor 8 participants reported using complementary and alternative medicine after diagnosis. This may suggest that their desire for natural healing of their cancer did not fully materialise after refusing treatment, or possibly they did not view diet and lifestyle as part of CAM when reporting their CAM use.

### Implications

By elucidating eight perspectives that reveal the motivations behind some individuals’ refusal of treatment, our findings provide valuable insights into cancer treatment refusal [61]. These findings may assist practitioners and family members to better understand the perspectives behind an individual’s decision to refuse cancer treatment, especially when refusal diverges from optimal survival outcomes [4]. Enhancing practitioner’s understanding of cancer treatment refusal may improve cancer care [12]. It may be beneficial for practitioners to consider the behavioural motivations we identified when discussing cancer treatment with individuals diagnosed with cancer. For instance, recognising that financial concerns can motivate treatment refusal may encourage practitioners to assess an individual’s financial situation to make the most appropriate treatment recommendation for that individual [62]. We recommend that practitioners engage in open communication about the financial aspects of treatment with individuals with cancer to ensure any financial concerns can be expressed and addressed. Certain treatment refusal motivations identified in this study, such as prognosis, are often discussed between practitioners and individuals with cancer [58]. This underscores the need to prioritise effective practitioner communication in cancer care. To ensure practitioners communicate information in a comprehensible manner, they are encouraged to consult guidelines on the most effective ways to communicate with individuals about cancer treatment [54].

Investigating the behavioural motivations of cancer treatment refusal is a novel research area. We recommend future researchers consider further investigating the behavioural motivations of treatment refusal we identified. For instance, our finding that R/S can motivate treatment refusal highlights the need for further exploration of the relationship between R/S and cancer treatment refusal. We recommend future studies investigate whether specific religions or spiritualities increase the likelihood of refusal, as identifying these may help practitioners identify individuals more at risk of refusing cancer treatment.

### Strengths and limitations

This study is, to our knowledge, the first to apply Q-methodology to explore the behavioural motivations of treatment refusal among individuals diagnosed with cancer. A strength of Q-methodology is its ability to collect and analyse rich data through the rigour of a statistical analysis [63]. Subjectivity, the theoretical approach of Q-methodology, enabled us to capture sensitive and diverse insights into the perspectives of individuals, which is often challenging with cancer-related topics [18].

Previous studies on cancer treatment refusal often recruited participants with a specific cancer type who refused a particular treatment, such as individuals with endometrial cancer who refused surgery [2]. In contrast, our P-set included participants with varied cancer diagnoses and types of treatment refusal. This diversity may contribute additional perspectives that are relevant across varied cancer experiences. Although, this diversity may also be viewed as a limitation, as treatment options can vary dramatically between cancer types. For instance, individuals diagnosed with soft tissue sarcoma are often limited to surgery and radiotherapy, with other treatments offering inconclusive benefits [64]. Being diagnosed with a cancer type that has fewer treatment options may influence the behavioural motivations for treatment refusal, and this should be considered when interpreting our findings.

Due to the small size of our P-set compared to other treatment refusal studies [13], we are cautious about over-emphasising what the demographic characteristics of our P-set may mean. Stenner and Watts [65] recommend a maximum P-set size of 60 participants for Q-methodology studies—a guideline that future researchers investigating cancer experiences may wish to adopt to capture a more comprehensive representation of diverse consumer perspectives. Future research may also seek to further detail potential mediated and moderated relationships between demographic and clinical characteristics of the sample and treatment decisions.

We did not collect data on the duration of time between participants’ treatment refusal and any treatment they received. This meant we were unable to determine whether a participant refused all or some treatments recommended to them at the time of their last treatment refusal. We predict that the motivations underlying the refusal of all recommended treatments would differ from those underlying the refusal of some, but not all, recommended treatments. For future studies, we recommend that researchers collect data on the times participants received and refused treatments.

Furthermore, unlike other Q-methodology studies, we chose not to conduct interviews with participants after they completed the Q-sort [66]. As our study was online and time-limited, we believed interviews would be too time-consuming to conduct. We acknowledge that the absence of participant interviews may have limited the exploration of participant perspectives. In future, researchers conducting online Q-methodology studies should plan ample time for participant interviews, given the richness of interview data [66].

## Conclusion

Using Q-methodology, this study explored the complex behavioural motivations underlying treatment refusal in individuals diagnosed with cancer. Identified behavioural motivations of treatment refusal varied across participants in this study. Some refused treatment due to physical, emotional, mental, or financial concerns associated with treatment. Some believed they were unable to undergo treatment because of their age or physical health. Additional treatment refusal motivations identified were religious and spiritual beliefs and practices, and a preference for natural healing over conventional treatment. Cancer treatment refusal is often misunderstood [61]; therefore, studies like this are needed to further understand the motivations of treatment refusal. This study provides crucial insight into behavioural motivations of treatment refusal. To improve cancer care and potentially improve cancer outcomes, future research is needed to explicate the behavioural motivations of treatment refusal we identified in this study.

## Supplementary Information

Below is the link to the electronic supplementary material.

Supplementary file1 (PDF 250 KB)

## Data Availability

No datasets were generated or analysed during the current study.
