# Supplementary material for: Exploring behavioural motivations of treatment refusal in cancer: a Q-methodological approach
Source: Support Care Cancer. 2025 Jul 8;33(8):667. doi: 10.1007/s00520-025-09666-5 (PMC12238116; doi:10.1007/s00520-025-09666-5)
Supplement: Supplementary file 1 — Supplementary file1 (PDF 250 KB) [file 520_2025_9666_MOESM1_ESM.pdf]

SUPPLEMENTARY MATERIALS

Supplementary Table 1 Unrotated Factor Matrix

**Factor**	**Eigenvalue**	**% Explained Variance**	**Cumulative % Explained Variance**
1	7.61	20	20
2	3.35	9	29
3	3.33	9	38
4	2.94	8	46
5	2.56	7	53
6	2.11	5	58
7	1.83	5	63
8	1.56	4	67

Supplementary Table 2 Characteristics of Factor 1 Participants

Factor characteristics		Number of participants
Gender	Female	1
	Male	5
Age [a]	Mean 52.8	*SD* 8.2
Age at treatment refusal	Mean 49.5	*SD* 8.9
Ethnicity	Black/African American	5
	African	1
	White American	1
	Did not disclose	1
Country	UK	5
	US	1
Conventional cancer treatment refused	Chemotherapy	3
	Radiotherapy	3
	Targeted therapy	2
Conventional cancer treatment received [b]	Chemotherapy	3
	Immunotherapy	3
	Radiotherapy	3
	Surgery	3
	Targeted therapy	2
	Hormone therapy	1
	Stem cell transplant	1
CAM received [b]	Yes	1
	No	5
Cancer status [a]	Cancer-free after treatment	3
	Cancer recurrence	2
	Remission	1

Note. CAM = Complementary and alternative medicine.

[a] Represents participants answers at the time of testing. [b] Received by participants at some point in time.

Supplementary Table 3 Characteristics of Factor 2 Participants

Factor characteristics		Number of participants
Gender	Female	3
	Male	1
Age [a]	Mean 44.5	*SD* 8.2
Age at treatment refusal	Mean 39.5	*SD* 10
Ethnicity	Black/African American	1
	Non-Indigenous Australian	1
	White American	1
	White European	1
Country	UK	2
	US	1
	Australia	1
Conventional cancer treatment refused	Radiotherapy	3
	Chemotherapy	1
	Surgery	1
Conventional cancer treatment received [b]	Surgery	2
	Radiotherapy	1

Factor characteristics		Number of participants
	Hormone therapy	1
CAM received	Yes	2
	No	2
Cancer status [a]	Cancer-free after treatment	1
	Remission	1
	Still in process of diagnosis	1
	Undergoing active treatment	1

Note. CAM = Complementary and alternative medicine.

[a] Represents participants' answers at the of testing. [b] Received by participant at some point in time.

Supplementary Table 4 Characteristics of Factor 3 Participants

Factor characteristics		Number of participants
Gender	Female	1
	Male	2
Age [a]	Mean 61.2	*SD* 5.8
Age at treatment refusal	Mean 61.2	*SD* 5.8
Ethnicity	White European	2
	Aboriginal and/or Torres Strait Islander	1
	White American	1
Country	US	3
	UK	1
Conventional cancer treatment refused	Chemotherapy	2
	Hormone therapy	1
Conventional cancer treatment received [b]	Surgery	2
	Radiation	1
CAM received [b]	Yes	1
	No	2
Cancer status [a]	Remission	2
	Still in process of diagnosis	1

Note. CAM = Complementary and alternative medicine.
[a] Represents participants at the time of testing. [b] Received by participants at some point in time.

Supplementary Table 5 Characteristics of Factor 4 Participants

Factor characteristics		Number of participants
Gender	Female	2
	Male	3
Age [a]	Mean 43.5	*SD* 11.1
Age at treatment refusal	Mean 39.5	*SD* 10
Ethnicity	Black/African American	2
	Hispanic	2
	Mixed	1
Country	US	4
	Australia	1
Conventional cancer treatment refused	Immunotherapy	2
	Surgery	2
	Chemotherapy	1
	Hormone therapy	1
	Radiotherapy	1
Conventional cancer treatment received [b]	Hormone therapy	3
	Chemotherapy	1
	Immunotherapy	1
CAM received [b]	Yes	1
	No	4
Cancer status [a]	Cancer-free after treatment	3
	Remission	1
	Undergoing active treatment	1
	Did not disclose	1

Note. CAM = Complementary and alternative medicine.
[a] Represents participants at the time of testing. [b] Received by participants at some point in time.

Supplementary Table 6 Characteristics of Factor 5 Participants

Factor characteristics		Number of participants
Gender	Female	3
	Male	1
Age [a]	Mean 49.5	*SD* 14.1
Age at treatment refusal	Mean 47	*SD* 11
Ethnicity	White European	2
	White American	1
	Did not disclose	1
Country	UK	2
	US	2
Conventional cancer treatment refused	Radiotherapy	2
	Surgery	2
	Chemotherapy	1
Conventional cancer treatment received [b]	Surgery	2
	Chemotherapy	1
	Hormone therapy	1
	Immunotherapy	1
	Radiotherapy	1
	Stem cell transplant	1
	Targeted therapy	1
CAM received [b]	Yes	1
	No	3
Cancer status [a]	Cancer-free after treatment	2
	Remission	1
	Did not disclose	1

Note. CAM = Complementary and alternative medicine.

[a] Represents participants at the time of testing. [b] Received by participants at some point in time.

Supplementary Table 7 Characteristics of Factor 6 Participants

Factor characteristics		Number of participants
Gender	Female	3
	Male	1
Age [a]	Mean 44.5	*SD* 10
Age at treatment refusal	Mean 42	*SD* 12.6
Ethnicity	White European	2
	Aboriginal and/or Torres Strait Islander	1
	White American	1
Country	US	3
	UK	1
Conventional cancer treatment refused	Chemotherapy	2
	Radiotherapy	2
	Surgery	2
	Stem cell transplant	1
Conventional cancer treatment received [b]	Surgery	3
	Chemotherapy	1
CAM received [b]	Yes	1
	No	3
Cancer status [a]	Remission	2
	Cancer recurrence	1
	Undergoing further diagnosis	1

Note. CAM = Complementary and alternative medicine.

[a] Represents participants at the time of testing. [b] Received by participants at some point in time.

Supplementary Table 8 Characteristics of Factor 7 Participants

Factor characteristics		Number of participants
Gender	Female	8
Age [a]	Mean 53.3	*SD* 16.0
Age at treatment refusal	Mean 44.5	*SD* 17.7
Ethnicity	Hispanic	3

	White American	3
	White European	2
Country	US	5
	UK	3
Conventional cancer treatment refused	Chemotherapy	3
	Surgery	2
	Hormone therapy	1
	Immunotherapy	1
	Radiotherapy	1
Conventional cancer treatment received [b]	Surgery	6
	Radiotherapy	3
	Chemotherapy	2
	Hormone therapy	2
	Immunotherapy	2
	Targeted therapy	2
	Stem cell transplant	1
CAM received [b]	Yes	2
	No	6
Cancer status [a]	Remission	3
	Cancer-free after treatment	3
	Palliative care	1
	Undergoing active treatment	1

Note. CAM = Complementary and alternative medicine.

[a] Represents participants at the time of testing. [b] Received by participants at some point in time.

Supplementary Table 9 Characteristics of Factor 8 Participants

Factor characteristics		Number of participants
Gender	Female	4
	Male	1
Age [a]	Mean 43.5	*SD* 11.4
Age at treatment refusal	Mean 43.5	*SD* 11.4
Ethnicity	White American	2
	Non-Indigenous Australian	1
	Mixed	1
	Did not disclose	1
Country	US	3
	Australia	2
Conventional cancer treatment refused	Chemotherapy	3
	Hormone therapy	2
	Radiotherapy	1
	Surgery	1
	Targeted therapy	1
Conventional cancer treatment received [b]	Chemotherapy	2
	Surgery	2
	Hormone therapy	2
	Immunotherapy	1
	Radiotherapy	1
	Targeted therapy	1
CAM received [b]	Yes	2
	No	3
Cancer status [a]	Remission	2
	Undergoing active treatment	2
	Still in process of diagnosis	1

Note. CAM = Complementary and alternative medicine.

[a] Represents participants at the time of testing. [b] Received by participants at some point in time.

Supplementary Table 10 Q-Sort Statements

Number	Content
1	I believed my poor health made conventional cancer treatment too risky.
2	I believed my other medical conditions made conventional cancer treatment too risky.
3	I believed that conventional cancer treatment was not tailored to my individual needs.
4	I believed that conventional cancer treatment would be detrimental to my emotional health.
5	I believed that conventional cancer treatment would be detrimental to my mental health.
6	I believed that conventional cancer treatment would not successfully treat my cancer, as it had not done so before.
7	I believed that conventional cancer treatment would not cure my cancer, instead it would only prolong my suffering.
8	I believed that I could manage my cancer through diet and lifestyle changes.
9	I believed that my age made conventional cancer treatment less beneficial and/or unnecessary.
10	I believed that my body could heal itself without the need for conventional cancer treatment.
11	I believed that participating in religious practices (e.g., praying) would heal my cancer.
12	I believed that participating in spiritual practices (e.g., manifestation) would heal my cancer.
13	I believed that the prognosis I was given if I was to have conventional cancer treatment was not good enough for me to choose to do it.
14	I believed the side effects of conventional cancer treatment would significantly reduce my quality of life.
15	I did not have enough support to assist me if I underwent conventional cancer treatment.
16	I did not trust the medical system, including the practitioners that recommended I underwent conventional cancer treatment.
17	I did not want my loved ones to have to support me throughout conventional cancer treatment.
18	I had more trust in alternative treatment experts than in conventional practitioners (e.g., doctors, oncologists).
19	I had previous negative experiences with medical treatments and/or hospitals.
20	I had seen others undergo conventional cancer treatment before, and I did not want to experience what they had.
21	I valued quality of life over quantity of life.
22	I wanted to avoid the hospital environment and remain in a more familiar setting.
23	I wanted to avoid the stigma and social impact of being a cancer patient.
24	I wanted to heal the cancer in my body naturally.
25	I wanted to undergo alternative cancer treatment rather than conventional cancer treatment.
26	I wanted to have more control over my treatment decisions.
27	I wanted to live my remaining time without the interference of conventional cancer treatment.
28	I wanted to maintain as much of my normal routine and activities as possible.
29	I was concerned about the financial burden of conventional cancer treatment.
30	I was concerned about the potential long-term side effects of conventional cancer treatment.
31	I was concerned about the uncertainty and unpredictability of conventional cancer treatment outcomes.
32	I was concerned that I would lose my independence if I underwent conventional cancer treatment.
33	I was concerned that my body would change forever if I underwent conventional cancer treatment.
34	I was happy with the life I had already lived, so I did not feel the need for prolonging my life with conventional cancer treatment.
35	I was influenced by my loved ones who supported alternative cancer treatment.
36	I was influenced by stories of people who had bad experiences with conventional cancer treatment.
37	I was influenced by stories of people who had treated their cancer with alternative cancer treatments.
38	I was overwhelmed by the complexity of conventional cancer treatment options.
39	I was sceptical about the effectiveness of conventional cancer treatment.
40	I was too tired from fighting my cancer to undergo more conventional cancer treatment.
41	My cultural beliefs influenced my decision to decline conventional cancer treatment.
42	My personal values and beliefs influenced my decision to decline conventional cancer treatment.
43	My religious beliefs influenced my decision to decline conventional cancer treatment.
44	My spiritual beliefs influenced my decision to decline conventional cancer treatment.
